# Caregiver Perceptions of Children’s Psychological Well-being During the COVID-19 Pandemic

**DOI:** 10.1001/jamanetworkopen.2021.11103

**Published:** 2021-04-29

**Authors:** Tali Raviv, Christopher M. Warren, Jason J. Washburn, Madeleine K. Kanaley, Liga Eihentale, Hayley Jane Goldenthal, Jaclyn Russo, Caroline P. Martin, Lisa S. Lombard, Jamie Tully, Kenneth Fox, Ruchi Gupta

**Affiliations:** 1 Center for Childhood Resilience, Pritzker Department of Psychiatry and Behavioral Health, Ann & Robert H. Lurie Children’s Hospital of Chicago, Chicago, Illinois; 2 Department of Psychiatry and Behavioral Sciences, Feinberg School of Medicine, Northwestern University, Chicago, Illinois; 3 Center for Food Allergy and Asthma Research, Institute for Public Health and Medicine, Feinberg School of Medicine, Northwestern University, Chicago, Illinois; 4 Academic General Pediatrics, Ann & Robert H. Lurie Children’s Hospital of Chicago, Chicago, Illinois; 5 Chicago Public Schools, Chicago, Illinois

## Abstract

**Importance:**

Understanding youth well-being during the COVID-19 pandemic can help appropriately allocate resources and inform policies to support youth.

**Objective:**

To examine caregiver-reported changes in the psychological well-being of their children 3 to 4 months after the start of COVID-19 stay-at-home orders, and to examine the association of caregiver-reported COVID-19 exposure and family stressors with caregiver perceptions of child psychological well-being.

**Design, Setting, and Participants:**

This survey study used an anonymous survey distributed via email from June 24 to July 15, 2020, to 350 000 families of students attending public schools in Chicago, Illinois. The a priori hypotheses were that caregivers would report worsening in child psychological well-being during the closure period compared with preclosure and that exposure to COVID-19–related stressors would be associated with a higher probability of worsening child psychological well-being. Data were analyzed from September 10, 2020, to March 15, 2021.

**Main Outcomes and Measures:**

Outcomes were 7 mental health concerns and 5 positive adjustment characteristics reported by caregivers using a retrospective pre-post design. COVID-19 exposure and family stressors were also reported by caregivers.

**Results:**

Among 350 000 families invited to participate, 32 217 caregivers (10 827 [39.3%] White, 8320 [30.2%] Latinx, 6168 [22.4%] Black; 2223 [8.1%] with multiple or other races/ethnicities) completed the survey on behalf of 49 397 children in prekindergarten through 12th grade. Child-specific outcomes were reported for 40 723 to 40 852 children depending on the specific question. The frequency of caregiver endorsement of youth mental health concerns ranged from 0.1 percentage point (suicidal ideation or self-harm, reported by 191 caregivers [0.5%] preclosure vs 246 caregivers [0.6%] during closure; *P* < .001) to 28.3 percentage points (loneliness, reported by 1452 caregivers [3.6%] preclosure vs 13 019 caregivers [31.9%] during closure; *P* < .001) higher after the end of in-person instruction compared with preclosure. Frequency of caregiver endorsement of youth positive adjustment characteristics ranged from −13.4 percentage points (plans for the future, reported by 18 114 caregivers [44.3%] preclosure vs 12 601 caregivers [30.9%] during closure; *P* < .001) to −30.9 percentage points (positive peer relationships, reported by 24 666 caregivers [60.4%] preclosure vs 19 130 caregivers [46.8%] during closure; *P* < .001) lower after the end of in-person instruction. Significant differences in COVID-19 exposure were observed across racial/ethnic (F_3,27 534_ = 614.8; *P* < .001) and household income strata (F_5,27 506_ = 842.0; *P* < .001). After accounting for covariates, all mental health concerns increased in probability (eg, angry: odds ratio, 1.55 [95% CI, 1.48-1.62]; *P* < .001) and all the positive adjustment characteristics decreased in probability (eg, hopeful or positive: odds ratio, 0.88 [95% CI, 0.84-0.92]; *P* < .001) as COVID-19 exposure and family stressors increased.

**Conclusions and Relevance:**

In this survey study of caregivers during the COVID-19 pandemic, COVID-19 and resulting exposure to stress were associated with worse youth psychological well-being, demonstrating the need for a comprehensive public health approach that prioritizes children’s well-being and draws broad public attention to the mental health needs of youth.

## Key Points

**Question:**

Is the COVID-19 pandemic and stressor exposure associated with caregivers’ perceptions of children’s psychological well-being?

**Findings:**

In this survey study among 32 217 caregivers of public-school students, endorsement of child mental health concerns was significantly higher and endorsement of positive adjustment characteristics was significantly lower after the end of in-person instruction compared with before. After accounting for covariates, child mental health concerns increased in probability and positive adjustment characteristics decreased in probability as COVID-19 exposure and family stressors increased.

**Meaning:**

These findings suggest that COVID-19 was associated with negative caregiver perceptions of children’s psychological well-being, requiring a comprehensive public health strategy.

## Teaser

This survey study examines the associations of school closure and exposure to COVID-19–related stressors with caregivers’ perceptions of their children’s mental well-being.

## Introduction

The COVID-19 pandemic is associated with stressors that have deleterious impacts on children’s mental health, including increased exposure to illness and death; economic, educational and social sequelae that result from stay-at-home orders; school closures and remote learning; and worsening caregiver mental health.^1,2,3,4,5,6^ Racially/ethnically marginalized youth, such as Black and Latinx individuals, and youth living in poverty have been disproportionately exposed to these stressors.^7,8^ Simultaneously, access to factors with protective associations, including positive school engagement and access to health and mental health resources, has been disrupted owing to efforts to reduce community transmission of COVID-19.^2,5,6,9^

Despite these concerns, few studies have examined youth mental health during the COVID-19 pandemic. Studies from early in the pandemic show an increase in depressive and anxiety symptoms among adolescents.^10,11^ A US national survey conducted by Patrick et al^12^ in June 2020 found that 26.9% of parents surveyed reported worsening of their own mental health, and 14.3% of parents reported worsening of their children’s behavioral health. Of note, the 10% of families in this sample who reported simultaneous worsening in parental mental health and children’s behavioral health indicated high rates of exposure to COVID-19–related stressors, such as the loss of childcare, food insecurity, and disruption in health care. A study by Gassman-Pines et al^13^ of hourly service workers with young children showed that even in the first weeks after implementation of pandemic restrictions, families experiencing more COVID-19–related financial stressors reported worsening psychological well-being for themselves as well as their children.

Less is known about risk for serious mental health concerns during the pandemic, such as suicide. In the first 3 months of school closures in Japan, no differences in suicide rates for youth younger than age 20 years were found compared with the same timeframe in 2018 and 2019.^14^ In contrast, significant increases in children’s self-reported symptoms of depression, self-harm, suicidal ideation, plans, and attempts were found immediately after school-reopening in China compared with immediately prior to the outbreak of the pandemic.^15^ An analysis by Leeb et al^16^ reported that although the overall number of mental health–related emergency department visits declined from March to May of 2020, the overall proportion of emergency department visits related to mental health concerns increased by 24% for children and by 31% for adolescents from March to October 2020 compared with the same time period in 2019.

This survey study used survey data from a large sample of caregivers whose children attended public school in Chicago, Illinois, to examine 2 hypotheses: that caregivers would report worsening in the psychological well-being of their children 3 to 4 months after the start of stay-at-home orders and resulting school closures and that exposure to COVID-19–related stressors would be associated with a higher probability of caregiver-reported worsening in child psychological well-being.

## Methods

The research review board at the Chicago Public Schools and Northwestern University’s institutional review board approved all study activities and exempted the study from written and oral consent because the study was anonymous and low risk.

### Procedure

The data for this study are from a cross-sectional survey examining caregiver attitudes about returning to in-person instruction after remote learning during the first phase of the COVID-19 pandemic in the United States. The survey was developed by a multidisciplinary team. Expert panel review was conducted, and the draft survey was pretested to ensure clarity, validity, and reliable functioning of all questions and response options before dissemination. Individuals were eligible to participate if they were a caregiver of a current student in the school district and were aged at least 18 years. For recruitment, the school district publicly distributed an email invitation and survey link to families that represent 350 000 students. The survey was offered in English, Polish, Spanish, and Chinese, the 4 most widely spoken languages among families in the school district.

The school district identified a subset of 171 127 students from 365 schools with a less than 5% response rate or without a parent email on file. From this subset, the research team attempted telephone outreach to 179 additional families. Of these, 22 families completed the survey via telephone. Families who did not participate did not have working telephone numbers (59 families), declined to take the survey (38 families), were left voicemails (29 families), or were otherwise ineligible to participate (eg, no longer had a child enrolled in the school district or needed additional translation not covered in the survey) (31 families).

Caregivers were asked to complete the survey regarding 1 of their children in the school system with the option to complete the survey for additional children. To document disparities shaped by structural racism, caregivers were asked to self-report on race/ethnicity using preset options or indicate they preferred not to answer. The survey remained open for responses from June 24, 2020, to July 15, 2020. The deidentified data set was shared with the research team by the school district for analyses.

### Measures

#### COVID-19 Exposure and Family Impact

The survey included 20 out of 25 items from the COVID-19 Exposure and Family Impact Scale (CEFIS).^17^ We omitted 5 items because nearly all participants were expected to respond affirmatively (eg, “We had a ‘stay-at-home’ order” or “Our schools / child care centers were closed”). A total CEFIS was derived by summing the number of specific CEFIS indicators selected by participants with complete data on all 20 items.

#### Psychological Well-being

Items assessing psychological well-being were developed specifically for this survey using a retrospective pre-post design.^18^ Parents were asked, “Which of the following generally describe your child BEFORE the end of in-person instruction on March 17, 2020?” Response options included 7 mental health concerns (ie, agitated or angry, lonely, anxious, stressed, depressed or low mood, self harm, and thoughts of suicide) and 5 positive adjustment characteristics (ie, relaxed, interacts positively with siblings or family members, has positive social or peer relationships, talks about plans for the near or far future [eg, summer, returning to school, college, work], and hopeful or positive). A follow-up question asked caregivers to indicate “Which of the following generally describe your child SINCE the end of in-person instruction on March 17, 2020?” Response options included the same 7 mental health concerns and 5 positive adjustment characteristics. Respondents were instructed to select all that applied to their child. Owing to low base rates, items assessing self-harm and thoughts of suicide were combined for analyses.

### Statistical Analysis

Summary descriptive statistics were calculated using R statistical software version 4.0 (R Project for Statistical Computing), which included raw counts and percentages for categorical variables, and means with SDs for CEFIS scores. Pairwise deletion of individuals with missing data on specific variables was used for all other analyses. This strategy leverages all available data and does not entirely omit individuals with missing data for some, but not all, variables of interest. One-way analysis of variance was used to test whether continuous CEFIS scores were significantly different across racial/ethnic and household income strata. A series of multiple logistic regression models were fit with the *lrm* command from the *rms* R package to better understand associations between CEFIS score and caregiver-reported changes in specific mental health concerns and positive adjustment characteristics in the child. Caregiver-reported presence or absence of each characteristic since the end of in-person instruction (ie, closure period) was modeled as the outcome. Caregiver-reported presence or absence of each specific characteristic preclosure was included as a covariate, along with city region (ie, north, south, central, and southwest). Given racial/ethnic and income disparities in exposure to COVID-19,^7,8,19^ race/ethnicity and household income were also included as covariates. Cluster robust SEs were specified at the household level to account for clustering of multiple children per household using the *robcov()* function, which implements the Huber-White method. Marginal effects at the mean of each covariate adjusted model were derived and visualized via ggpredict, from the ggeffects package in R.

*P* values were 2-sided, and statistical significance was set at *P* = .05. Data were analyzed from September 10, 2020, to March 15, 2021.

## Results

Surveys were completed by a racially/ethnically diverse sample of 32 217 caregivers, including 10 827 White caregivers (39.3%), 8320 Latinx caregivers (30.2%), 6168 Black caregivers (22.4%), and 2223 caregivers (8.1%) with multiple or other races/ethnicities (Table 1). Approximately half of caregivers reported on 1 student (16 007 caregivers (49.7%), 11 863 caregivers (36.8%) reported on 2 students, and 4347 caregivers (13.5%) reported on 3 or more students within their household, for a total of 49 397 children (Table 1). Child-specific outcomes were reported on 40 723 to 40 852 children, depending on the specific question.

**Table 1.** Participant Demographic Characteristics

Characteristic	No. (%) (N = 32 217)
Race/ethnicity	
Black	6168 (22.4)
Latinx	8320 (30.2)
White	10 827 (39.3)
Multiple or other	2223 (8.1)
Prefer not to answer	4679 (14.5)
Survey language	
Chinese	266 (0.8)
English	29 889 (92.8)
Polish	113 (0.4)
Spanish	1949 (6.0)
Annual household income, $	
<20 000	2973 (10.8)
20 000-34 999	3830 (13.9)
35 000-49 999	3237 (11.8)
50 000-74 999	4025 (14.6)
75 000-99 999	3782 (13.8)
≥100 000	9665 (35.1)
Prefer not to answer	4705 (14.6)
No. of children attending school in the district	
1	16 007 (49.7)
2	11 863 (36.8)
3	3377 (10.5)
4	736 (2.3)
≥5	234 (0.7)
Children's grades	
Prekindergarten	1806 (3.7)
Kindergarten	3356 (6.8)
1	4243 (8.6)
2	4077 (8.3)
3	3995 (8.1)
4	3715 (7.5)
5	3658 (7.4)
6	3520 (7.1)
7	3551 (7.2)
8	3114 (6.3)
9	3896 (7.9)
10	3929 (8.0)
11	3422 (6.9)
12	3115 (6.3)
Individualized Educational Plan^a^	
Yes	5373 (13.1)
No	35 479 (86.9)
504-plan^b^	
Yes	4189 (10.3)
No	36 663 (89.7)

^a^ An Individualized Educational Plan is a legal document indicating necessary supports and services for children eligible for special education.

^b^ A 504 Plan is a legal document indicating school accommodations addressing how a child with a specified disability can learn.

Frequency of endorsement of each of the 6 mental health concerns was significantly greater when respondents were prompted to describe their child during closure compared with preclosure (Table 2). The frequency of caregiver endorsement of youth mental health concerns ranged from 0.1 percentage point (suicidal ideation or self-harm, reported by 191 caregivers [0.5%] preclosure vs 246 caregivers [0.6%] during closure; *P* < .001) to 28.3 percentage points higher (loneliness, reported by 1452 caregivers [3.6%] preclosure vs 13 019 caregivers [31.9%] during closure; *P* < .001) (Table 2). In contrast, frequency of endorsement of each of the 5 positive adjustment characteristics was significantly lower when respondents were prompted to describe their child during closure compared with preclosure. Frequency of caregiver endorsement of youth positive adjustment characteristics ranged from −13.4 percentage points (plans for the future, reported by 18 114 caregivers [44.3%] preclosure vs 12 601 caregivers [30.9%] during closure; *P* < .001) to −30.9 percentage points lower (positive peer relationships, reported by 24 666 caregivers [60.4%] preclosure vs 19 130 caregivers [46.8%] during closure; *P* < .001) (Table 2). Pearson χ^2^ testing found each of the pre-post differences in the frequency of endorsing all 6 mental health concerns and 5 positive adjustment characteristics to be statistically significant. eTable 1 in the Supplement displays the frequencies of endorsing mental health concerns and positive adjustment characteristics by family income level.

**Table 2.** Caregiver Reports of Child Mental Health and Positive Adjustment Characteristics for All Participants and by Race/Ethnicity

Characteristic	No. (%)							
	All participants		Race/ethnicity					
			Black		Hispanic/Latinx		White	
	Before^a^	Since^b^	Before^a^	Since^b^	Before^a^	Since^b^	Before^a^	Since^b^
Agitated or angry	1712 (4.2)	9752 (23.9)	393 (5.3)	1107 (14.9)	494 (4.5)	1720 (15.8)	486 (3.5)	4913 (35.8)
Anxious	5137 (12.6)	9497 (23.3)	648 (8.7)	1132 (15.2)	1157 (10.6)	1939 (17.8)	2324 (16.9)	4478 (32.6)
Depressed or low mood	1387 (3.4)	5715 (14.0)	272 (3.7)	612 (8.2)	359 (3.3)	906 (8.3)	460 (3.4)	3006 (21.9)
Lonely	1452 (3.6)	13 019 (31.9)	255 (3.4)	1708 (22.9)	380 (3.5)	1959 (17.9)	470 (3.4)	6637 (48.4)
Stressed	4773 (11.7)	9957 (24.4)	734 (9.9)	1265 (17.0)	1281 (11.7)	2106 (19.3)	1707 (12.4)	4479 (32.6)
Self-harm or thoughts of suicide	191 (0.5)	246 (0.6)	33 (0.5)	29 (0.4)	47 (0.4)	32 (0.3)	25 (0.9)	30 (1.0)
Had positive social or peer relationships	26 995 (66.1)	14 386 (35.2)	4667 (62.7)	2838 (38.1)	5770 (52.8)	3377 (30.9)	10 861 (79.1)	5250 (38.3)
Hopeful or positive	20 052 (49.1)	12 012 (29.4)	3709 (49.8)	2712 (36.4)	4393 (40.2)	3357 (30.7)	7638 (55.7)	3383 (24.6)
Interacted positively with siblings or family	24 666 (60.4)	19 130 (46.8)	4100 (55.0)	3550 (47.7)	5473 (50.1)	4552 (41.7)	9896 (72.1)	7035 (51.3)
Relaxed	21 414 (52.4)	15 056 (36.9)	3987 (53.5)	3276 (44)	5887 (53.9)	4644 (42.5)	6973 (50.8)	3829 (27.9)
Talks about plans for the future	18 114 (44.3)	12 601 (30.9)	3169 (42.5)	2628 (35.3)	3814 (34.9)	2956 (27.1)	7392 (53.9)	4440 (32.3)

^a^ Before the end of in-person instruction on March 17, 2020.

^b^ Since the end of in-person instruction on March 17, 2020.

Between 5236 youths (12.8%) and 12 351 youths (30.2%) were described by caregivers as angry, anxious, depressed, lonely, or stressed during closure, but not preclosure (eTable 2 in the Supplement). Similarly, between 8623 youths (21.1%) and 18 114 youths (44.3%) were described by their caregivers as exhibiting the positive adjustment characteristics preclosure, but not during closure. Very few youths were described by their caregivers as having specific mental health concerns preclosure but not during closure (eTable 2 in the Supplement). However, some youths were described by their caregivers as having new positive adjustment characteristics since the end of in-person instruction (eTable 2 in the Supplement).

Covariate-adjusted probability of parental endorsement of the mental health concerns and positive adjustment characteristics during closure varied by race/ethnicity and income. eTable 3 in the Supplement displays the estimated odds ratios (ORs) and corresponding 95% CIs for model parameters estimating each parent-endorsed child characteristic. Caregivers who identified as non-Latinx White were significantly more likely to endorse each mental health concern and significantly less likely to endorse each positive adjustment characteristic during closure compared with caregivers who identified as Black or Latinx. With respect to household income, covariate-adjusted probability of parental endorsement of each mental health concern during closure increased as household income increased.

Table 3 summarizes the CEFIS scores. One-way analysis of variance revealed significant differences in overall CEFIS scores across racial/ethnic (F_3,27534_ = 614.8; *P* < .001) and household income strata (F_5,27506_ = 842.0; *P* < .001). Across the entire sample, respondents reported a mean (SD) CEFIS score of 2.5 (2.2). Mean (SD) CEFIS scores were higher among Black (2.8 [2.4]) and Latinx (3.1 [2.3]) respondents compared with non-Latinx White respondents (1.8 [1.8]) (Table 3). Significantly greater exposure was identified by Black or Latinx respondents vs White respondents for all items except “We had difficulty getting health care when we needed it,” “A member of the family had to cut back hours at work,” and “Our family income decreased.” Mean (SD) CEFIS scores were higher among the lowest-earning households (3.7 [2.7]) vs the highest-earning households (1.6 [1.6]), and the highest earning households were less likely to report each specific COVID experience except for difficulty getting healthcare (eTable4 in the Supplement).

**Table 3.** COVID-19 Exposure and Family Impacts for All Participants and by Race/Ethnicity

COVID-19 family exposure	No. (%)			
	All participants	Race/ethnicity		
		Black	Hispanic/Latinx	White
Stopped working temporarily	6074 (18.9)	1205 (19.5)	2115 (25.4)	1487 (13.7)
Permanently lost job	2688 (8.3)	570 (9.2)	803 (9.7)	742 (6.9)
Kept working outside of home	14 208 (44.1)	2886 (46.8)	4606 (55.4)	3785 (35.0)
Health care practitioner	4052 (12.6)	896 (14.5)	835 (10.0)	1264 (11.7)
Cut back hours	7313 (22.7)	1114 (18.1)	2222 (26.7)	2389 (22.1)
Moved out of home	184 (0.6)	48 (0.8)	60 (0.7)	30 (0.3)
Lost health insurance	634 (2.0)	133 (2.2)	200 (2.4)	145 (1.3)
Family income decreased	10 577 (32.8)	1647 (26.7)	3257 (39.1)	3360 (31.0)
Difficulty				
Getting other essentials	4308 (13.4)	1296 (21.0)	1475 (17.7)	628 (5.8)
Getting medicine	1102 (3.4)	369 (6.0)	335 (4.0)	128 (1.2)
Getting health care	1728 (5.4)	450 (7.3)	497 (6.0)	326 (3.0)
Getting food	1881 (5.8)	571 (9.3)	661 (7.9)	239 (2.2)
Getting face masks, sanitizer, or other products	9461 (29.4)	2197 (35.6)	3268 (39.3)	2079 (19.2)
Could not pay				
Rent	1840 (5.7)	433 (7.0)	800 (9.6)	256 (2.4)
Bills	2496 (7.7)	694 (11.3)	988 (11.9)	325 (3.0)
Children took on job outside of home	275 (0.9)	57 (0.9)	101 (1.2)	44 (0.4)
Children assumed childcare responsibilities	1983 (6.2)	422 (6.8)	605 (7.3)	487 (4.5)
Someone in family				
Was exposed to COVID-19	4746 (14.7)	949 (15.4)	1469 (17.7)	1378 (12.7)
Had COVID-19 symptoms or was diagnosed	3407 (10.6)	829 (13.4)	1268 (15.2)	715 (6.6)
Died of COVID-19	1403 (4.4)	521 (8.4)	481 (5.8)	175 (1.6)
Overall CEFIS score, mean (SD)^a^	2.5 (2.2)	2.8 (2.4)	3.1 (2.3)	1.8 (1.8)

Abbreviation: CEFIS, COVID-19 Exposure and Family Impact Scale.

^a^ Range, 0 to 20, with higher scores indicating more exposure.

**Figure 1.**
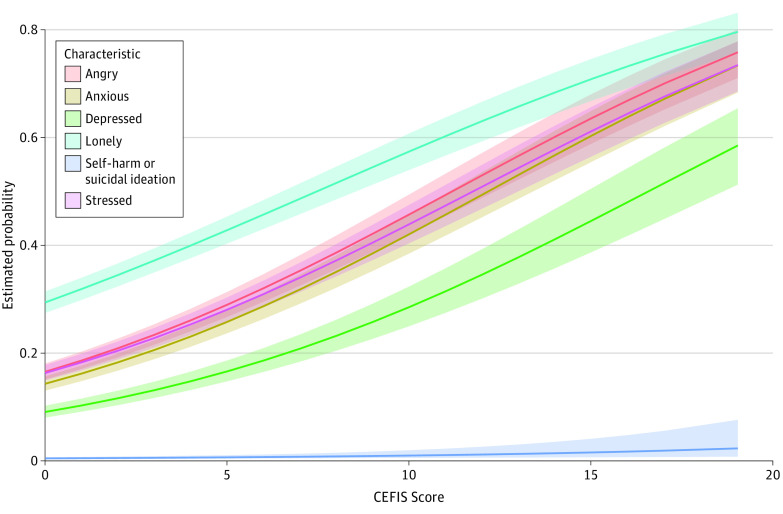
Adjusted Probabilities of Child Mental Health Concerns After the End of In-Person Instruction CEFIS indicates COVID-19 Exposure and Family Impact Scale.

**Figure 2.**
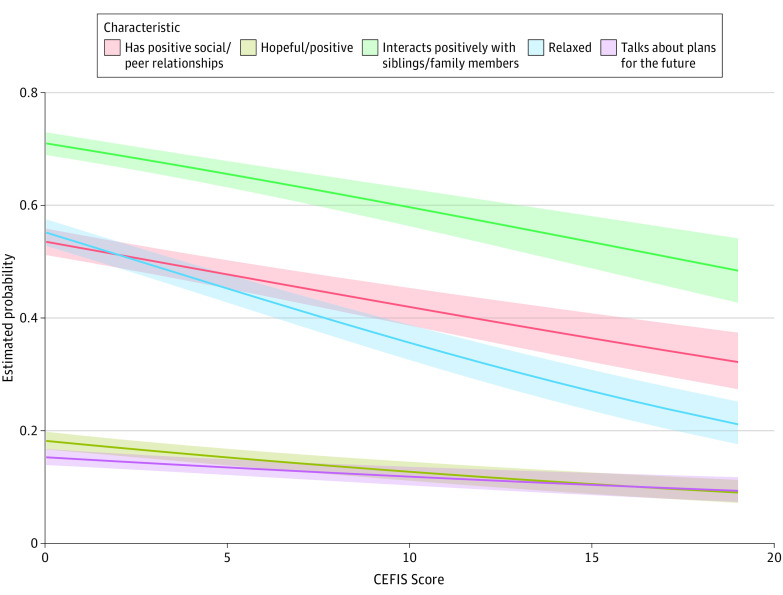
Adjusted Probabilities of Positive Adjustment Characteristics After the End of In-Person Instruction CEFIS indicates COVID-19 Exposure and Family Impact Scale.

Figure 1 and Figure 2 show the probability of each mental health concern and positive adjustment characteristic since the end of in-person instruction by CEFIS score after accounting for clustering of multiple children within a household and adjusting for endorsement preclosure, as well as participant race/ethnicity, household income, and zip code. All the mental health concerns significantly increased in probability (eg, angry: OR, 1.55 [95% CI, 1.48-1.62]; *P* < .001) and all the positive adjustment characteristics significantly decreased in probability (eg, hopeful or positive: OR, 0.88 [95% CI, 0.84-0.92]; *P* < .001) as CEFIS score increased (eTable 3 in the Supplement).

## Discussion

In this survey study, caregivers reported pervasive negative perceptions of their children’s psychological well-being associated with the COVID-19 pandemic. After the end of in-person instruction, nearly a quarter of children were described by their caregivers as angry or agitated, more than one-quarter of children were described by their caregivers as anxious and stressed, more than one-third were described as lonely, and fewer than one-third of caregivers reported that their child had positive peer relationships. Children’s anger and behavioral challenges influence caregiving stress^20^ and disrupt caregivers’ ability to regulate their own emotions and manage children’s behaviors.^21^ This transactional pattern has been associated with coercive parenting practices that may exacerbate children’s behavior problems^22^ and increase risk for child maltreatment.^23^ Increased loneliness may impact developing social skills among younger children and increase adverse health outcomes, poor academic performance, anxiety, depression, and suicide among adolescents.^24^

Rates of caregiver endorsement of children’s self-harm behaviors and suicidal ideation were low prior to and after school closure was enacted, with only a slight increase after closure. These findings should be interpreted with caution, as caregivers may be unaware of their children’s self-harming behavior or suicidal ideation. When surveyed directly, Centers for Disease Control and Prevention^25^ estimates indicate that 18.8% of students seriously considered suicide and 8.9% attempted suicide in the previous year. Given that youth suicide rates have increased by nearly 60% over the past 10 years,^26^ additional research is needed to better understand the impact of COVID-19 on suicidal ideation and behavior.

The findings of this survey study suggest that the COVID-19 pandemic is associated with widespread effects on children’s mental health, linked not only to children’s mood and peer relationships but also to their positive adjustment and familial interactions. For all youth, COVID-19 was associated with prolonged disruptions in daily routines, decreased exposure to adult and peer relationships, and reduced opportunities for academic and social development. Compounding these issues are the economic hardship, grief and loss, and health concerns that some youth have experienced as a result of COVID-19. In our sample, Black, Latinx, and youth living in poorer households were most likely to experience these additional stressors. Only 3 of 20 items on the CEFIS showed no significant differences by race/ethnicity, likely owing to the nearly universal consequences of widespread stay-at-home orders early in the pandemic. Given that non-Latinx White and wealthier households were more likely to report mental health concerns and less likely to report positive adjustment characteristics during closure, future research should examine differences in the processes of resilience among racially and ethnically marginalized households.

### Public Health Implications

Our findings indicate a need for a research-informed public health approach that prioritizes children’s well-being and mental health.^2,27^ Such approaches must include primary prevention efforts that target known risk factors likely to be caused or exacerbated by COVID-19, such as poverty, socioeconomic inequalities, health disparities, and stress. As our findings demonstrate, Black, Latinx, and low-income families reported disproportionately high rates of COVID-19 stressor exposure, likely attributable to systemic racism and ensuing structural inequities, such as underresourcing and lack of access to health care.^7^

Primary prevention efforts must be paired with targeted assessments to identify youth in need of support and policies that support equitable access to mental health care. New approaches are needed, such as brief and low-intensity interventions that can be implemented by individuals without specialized training.^1^ In addition, engaging youth and community members in policy and program development is critical to ensure that mental health promotion efforts are contextually and culturally relevant.^28^ Needs of communities vary widely, and funds should be directed and allocated in ways that will have the largest and most meaningful impact while not exacerbating existing inequities.

Prior to the pandemic, schools represented a major access point for food, health care, and mental health services.^29,30,31^ Schools have the potential to serve as hubs that support community healing and align public health strategies across child-serving systems. However, the public education system, already burdened by the need to adapt traditional models of education to the constraints of public health guidelines, will need additional support to respond in innovative ways to the increased mental health needs. There will also be a need to shore up access to school- and community-based behavioral health services, including tele–behavioral health services. Resources must also be provided to support educator health and well-being to address workplace stress and secondary traumatic stress among teachers.^32^ Finally, social justice requires that policy efforts are crafted to unmask, disrupt, and address disparities in COVID-19 needs and resources.^33^

### Limitations

This study has several limitations. First, data were collected from a sample of families residing in a large urban area and may not be representative of rural and suburban areas or areas with different COVID-19 exposure, social distancing policies, and resources. Furthermore, although the sample was sociodemographically diverse, it was not representative of families in the district. Based on publicly available district data, a higher percentage of survey respondents were White, English-speaking, and had higher income levels compared with district demographic characteristics.^34^ This response bias may lead to underestimates of the effects on psychological well-being, as it is possible that nonrespondents were experiencing higher levels of COVID-19–related stress than respondents. It is imperative that future research better engage racially marginalized families, low-income families, and immigrant and non-English speaking families to understand the true mental health impact of COVID-19 stressors. Second, the data rely exclusively on caregiver report. It has long been noted that cross-informant correlations on mental health concerns tend to be low to moderate,^35^ underscoring the importance of obtaining self-reports for youth. Third, this study used single-item, face valid questions to assess mental health concerns and positive adjustment rather than normed measures of mental health problems or adaptive functioning, limiting understanding of clinically significant distress or impairment. Fourth, school district leadership determined that the survey should be administered as rapidly as possible within a limited time to inform school district policy around school reopening. This necessitated the email distribution of deidentified survey links in lieu of individualized email invitations, thereby preventing the derivation of true survey response rates and raising the possibility—albeit unlikely—of multiple responses per household. The short survey field period also contributed to the very low rate of telephone follow-up. Additionally, the data were collected at a single point in time and rely on retrospective recall. As the COVID-19 pandemic continues, longitudinal data will help clarify whether initial distress is reduced, maintained, or intensified over time as well as identify subgroups of youth most at risk for continued concerns.

## Conclusions

The findings of this survey study suggest that COVID-19 and resulting exposure to stress were associated with negative caregiver perceptions of children’s psychological well-being. The prevalence of these concerns demonstrate the need for a comprehensive public health approach that prioritizes children’s well-being and draws broad public attention to the mental health needs of youth.

## Notes

Supplement. **eTable 1.** Caregiver Reports of Child Mental Health and Positive Adjustment Characteristics by Household Income Level **eTable 2.** Frequency of Caregiver-Reported Youth Mental Health Concerns and Positive Adjustment Characteristics Before and Since the End of In-person Instruction **eTable 3.** Covariate-Adjusted Probability of Parental Endorsement of Mental Health Concerns and Positive Adjustment Characteristics **eTable 4.** COVID-19 Exposure/Family Impacts by Household Income Level

## References

[1] Gruber J, Prinstein MJ, Clark LA, . Mental health and clinical psychological science in the time of COVID-19: Challenges, opportunities, and a call to action. Am Psychol. Published online August 10, 2020. doi:10.1037/amp0000707 32772538 PMC7873160

[2] Stark AM, White AE, Rotter NS, Basu A. Shifting from survival to supporting resilience in children and families in the COVID-19 pandemic: lessons for informing U.S. mental health priorities. Psychol Trauma. 2020;12(S1):S133-S135. doi:10.1037/tra0000781 32525375

[3] Janssen LHC, Kullberg MJ, Verkuil B, . Does the COVID-19 pandemic impact parents’ and adolescents’ well-being: an EMA-study on daily affect and parenting. PLoS One. 2020;15(10):e0240962. doi:10.1371/journal.pone.0240962 33064778 PMC7567366

[4] Marchetti D, Fontanesi L, Mazza C, Di Giandomenico S, Roma P, Verrocchio MC. Parenting-related exhaustion during the Italian COVID-19 lockdown. J Pediatr Psychol. 2020;45(10):1114-1123. doi:10.1093/jpepsy/jsaa093 33068403 PMC7665691

[5] Russell BS, Hutchison M, Tambling R, Tomkunas AJ, Horton AL. Initial challenges of caregiving during COVID-19: caregiver burden, mental health, and the parent-child relationship. Child Psychiatry Hum Dev. 2020;51(5):671-682. doi:10.1007/s10578-020-01037-x 32749568 PMC7398861

[6] Xu Y, Wu Q, Levkoff SE, Jedwab M. Material hardship and parenting stress among grandparent kinship providers during the COVID-19 pandemic: the mediating role of grandparents’ mental health. Child Abuse Negl. 2020;110(Pt 2):104700-104700. doi:10.1016/j.chiabu.2020.104700 32854948 PMC7444952

[7] Moreno C, Wykes T, Galderisi S, . How mental health care should change as a consequence of the COVID-19 pandemic. Lancet Psychiatry. 2020;7(9):813-824. doi:10.1016/S2215-0366(20)30307-2 32682460 PMC7365642

[8] Purtle J. COVID-19 and mental health equity in the United States. Soc Psychiatry Psychiatr Epidemiol. 2020;55(8):969-971. doi:10.1007/s00127-020-01896-8 32556376 PMC7298157

[9] Golberstein E, Wen H, Miller BF. Coronavirus disease 2019 (COVID-19) and mental health for children and adolescents. JAMA Pediatr. 2020;174(9):819-820. doi:10.1001/jamapediatrics.2020.1456 32286618

[10] Duan L, Shao X, Wang Y, . An investigation of mental health status of children and adolescents in china during the outbreak of COVID-19. J Affect Disord. 2020;275:112-118. doi:10.1016/j.jad.2020.06.029 32658812 PMC7329661

[11] Racine N, Cooke JE, Eirich R, Korczak DJ, McArthur B, Madigan S. Child and adolescent mental illness during COVID-19: a rapid review. Psychiatry Res. 2020;292:113307-113307. doi:10.1016/j.psychres.2020.113307 32707216 PMC7363598

[12] Patrick SW, Henkhaus LE, Zickafoose JS, . Well-being of parents and children during the COVID-19 pandemic: a national survey. Pediatrics. 2020;146(4):e2020016824. doi:10.1542/peds.2020-016824 32709738

[13] Gassman-Pines A, Ananat EO, Fitz-Henley J II. COVID-19 and parent-child psychological well-being. Pediatrics. 2020;146(4):e2020007294. doi:10.1542/peds.2020-007294 32764151 PMC7546085

[14] Isumi A, Doi S, Yamaoka Y, Takahashi K, Fujiwara T. Do suicide rates in children and adolescents change during school closure in Japan: the acute effect of the first wave of COVID-19 pandemic on child and adolescent mental health. Child Abuse Negl. 2020;110(Pt 2):104680-104680. doi:10.1016/j.chiabu.2020.104680 32847679 PMC7443207

[15] Zhang L, Zhang D, Fang J, Wan Y, Tao F, Sun Y. Assessment of mental health of Chinese primary school students before and after school closing and opening during the COVID-19 pandemic. JAMA Netw Open. 2020;3(9):e2021482. doi:10.1001/jamanetworkopen.2020.21482 32915233 PMC7489803

[16] Leeb RT, Bitsko RH, Radhakrishnan L, Martinez P, Njai R, Holland KM. Mental health–related emergency department visits among children aged <18 years during the COVID-19 pandemic—United States, January 1–October 17, 2020. MMWR Morb Mortal Wkly Rep. 2020;69(45):1675-1680. doi:10.15585/mmwr.mm6945a3 33180751 PMC7660659

[17] Kazak A, Canter K, Phan-Vo T-L, . COVID-19 Exposure and Family Impact Survey (CEFIS). Accessed May 5, 2020. https://www.nlm.nih.gov/dr2/CEFIS_COVID_questionnaire_English_42220_final.pdf

[18] Little TD, Chang R, Gorrall BK, . The retrospective pretest–posttest design redux: on its validity as an alternative to traditional pretest–posttest measurement. Int J Behav Dev. 2020;44(2):175-183. doi:10.1177/0165025419877973

[19] Masonbrink AR, Hurley E. Advocating for children during the COVID-19 school closures. Pediatrics. 2020;146(3):e20201440. doi:10.1542/peds.2020-1440 32554517

[20] Barry TD, Dunlap ST, Cotten SJ, Lochman JE, Wells KC. The influence of maternal stress and distress on disruptive behavior problems in boys. J Am Acad Child Adolesc Psychiatry. 2005;44(3):265-273. doi:10.1097/00004583-200503000-00011 15725971

[21] Blair C, Granger DA, Willoughby M, ; FLP Investigators. Salivary cortisol mediates effects of poverty and parenting on executive functions in early childhood. Child Dev. 2011;82(6):1970-1984. doi:10.1111/j.1467-8624.2011.01643.x 22026915 PMC3218241

[22] Patterson GR. Coercion theory: the study of change. In: Dishion TJ, Snyder J, eds. The Oxford Handbook of Coercive Relationship Dynamics. Oxford University Press; 2016:7-22.

[23] Cicchetti D, Toth SL, Maughan A. An ecological-transactional model of child maltreatment. In: Sameroff AJ, Lewis M, Miller SM, eds. Handbook of Developmental Psychopathology. 2nd ed. Kluwer Academic Publishers; 2000:689-722. doi:10.1007/978-1-4615-4163-9_37

[24] Heinrich LM, Gullone E. The clinical significance of loneliness: a literature review. Clin Psychol Rev. 2006;26(6):695-718. doi:10.1016/j.cpr.2006.04.002 16952717

[25] Centers for Disease Control and Prevention. Youth Risk Behavior Surveillance System (YRBSS). Accessed December 15, 2020. https://www.cdc.gov/yrbs

[26] Curtin SC. State suicide rates among adolescents and young adults aged 10–24: United States, 2000–2018. *National Vital Statistics Reports*. 2020;69(11):1-10. Accessed December 16, 2020. https://www.cdc.gov/nchs/data/nvsr/nvsr69/nvsr-69-11-508.pdf 33054915

[27] Campion J, Javed A, Sartorius N, Marmot M. Addressing the public mental health challenge of COVID-19. Lancet Psychiatry. 2020;7(8):657-659. doi:10.1016/S2215-0366(20)30240-6 32531299 PMC7282758

[28] Jenkins E, Haines-Saah R, McGuinness L, . Assessing the impacts of the Agenda Gap intervention for youth mental health promotion through policy engagement: a study protocol. Int J Ment Health Syst. 2020;14(1):58. doi:10.1186/s13033-020-00390-7 32765643 PMC7395361

[29] Allison MA, Crane LA, Beaty BL, Davidson AJ, Melinkovich P, Kempe A. School-based health centers: improving access and quality of care for low-income adolescents. Pediatrics. 2007;120(4):e887-e894. doi:10.1542/peds.2006-2314 17846146

[30] Farmer EM, Burns BJ, Phillips SD, Angold A, Costello EJ. Pathways into and through mental health services for children and adolescents. Psychiatr Serv. 2003;54(1):60-66. doi:10.1176/appi.ps.54.1.60 12509668

[31] Fletcher JM, Frisvold DE. The relationship between the school breakfast program and food insecurity. J Consum Aff. 2017;51(3):481-500. doi:10.1111/joca.12163 30008484 PMC6040671

[32] Hydon S, Wong M, Langley AK, Stein BD, Kataoka SH. Preventing secondary traumatic stress in educators. Child Adolesc Psychiatr Clin N Am. 2015;24(2):319-333. doi:10.1016/j.chc.2014.11.003 25773327

[33] Hamilton LS, Kaufman JH, Diliberti M. Teaching and leading through a pandemic: key findings from the American Educator Panels Spring 2020 COVID-19 surveys. Accessed December 21, 2020. https://www.rand.org/pubs/research_reports/RRA168-2.html

[34] Chicago Public Schools. Demographics: racial/ethnic report. Accessed December 28, 2020. https://www.cps.edu/about/district-data/demographics/#a_racial-ethnic-report

[35] Achenbach TM, McConaughy SH, Howell CT. Child/adolescent behavioral and emotional problems: implications of cross-informant correlations for situational specificity. Psychol Bull. 1987;101(2):213-232. doi:10.1037/0033-2909.101.2.213 3562706

