# Supplementary material for: Caregiver Perceptions of Children’s Psychological Well-being During the COVID-19 Pandemic
Source: JAMA Netw Open. 2021 Apr 29;4(4):e2111103. doi: 10.1001/jamanetworkopen.2021.11103 (PMC8085728; doi:10.1001/jamanetworkopen.2021.11103)
Supplement: Supplement. — eTable 1. Caregiver Reports of Child Mental Health and Positive Adjustment Characteristics by Household Income Level eTable 2. Frequency of Caregiver-Reported Youth Mental Health Concerns and Positive Adjustment Characteristics Before and Since the End of In-person Instruction eTable 3. Covariate-Adjusted Probability of Parental Endorsement of Mental Health Concerns and Positive Adjustment Characteristics eTable 4. COVID-19 Exposure/Family Impacts by Household Income Level [file jamanetwopen-e2111103-s001.pdf]

Supplemental Online Content

Raviv T, Warren CM, Washburn JJ, et al. Caregiver perceptions of children's psychological well-being during the COVID-19 pandemic. *JAMA Netw Open.* 2021;4(4):e2111103. doi:10.1001/jamanetworkopen.2021.11103

eTable 1. Caregiver Reports of Child Mental Health and Positive Adjustment Characteristics by Household Income Level

eTable 2. Frequency of Caregiver-Reported Youth Mental Health Concerns and Positive Adjustment Characteristics Before and Since the End of In-person Instruction

eTable 3. Covariate-Adjusted Probability of Parental Endorsement of Mental Health Concerns and Positive Adjustment Characteristics

eTable 4. COVID-19 Exposure/Family Impacts by Household Income Level

This supplemental material has been provided by the authors to give readers additional information about their work.

© 2021 Raviv T et al. *JAMA Network Open.*

eTable 1. Caregiver Reports of Child Mental Health and Positive Adjustment Characteristics by Household Income Level

	**ANNUAL HOUSEHOLD INCOME**					
	**>$100,000, N. (%)**		**$75,000-$99,999, N. (%)**		**$50,000-$74,999, N. (%)**	
**Characteristic**	**Before[a]**	**Since[b]**	**Before[a]**	**Since[b]**	**Before[a]**	**Since[b]**
Agitated/Angry	382(3.1)	4105(33)	175(3.7)	1328(28)	203(4.1)	1092(21.9)
Anxious	1945(15.6)	3731(30)	634(13.4)	1275(26.9)	587(11.8)	1146(23)
Depressed/Low Mood	399(3.2)	2432(19.5)	141(3)	752(15.8)	163(3.3)	628(12.6)
Lonely	381(3.1)	5625(45.2)	161(3.4)	1800(37.9)	197(3.9)	1494(29.9)
Stressed	1487(12)	3729(30)	514(10.8)	1332(28.1)	563(11.3)	1178(23.6)
Self-harm OR Thoughts of suicide	55(0.5)	106(0.6)	22(0.5)	31(0.7)	32(0.8)	15(0.4)
Had positive social/peer relationships	10,051(80.8)	5050(40.6)	3466(73)	1677(35.3)	3269(65.5)	1766(35.4)
Hopeful/Positive	7121(57.2)	3516(28.3)	2521(53.1)	1377(29)	2449(49)	1543(30.9)
Interacted positively with siblings/family	9170(73.7)	6752(54.3)	3181(67)	2355(49.6)	2918(58.4)	2336(46.8)
Relaxed	6496(52.2)	3832(30.8)	2564(54)	3132(66)	2596(52)	1870(37.5)
Talks about plans for the future	6837(55)	4257(34.2)	2270(47.8)	1573(33.1)	2193(43.9)	1629(32.6)

[a] Before the end of in-person instruction on March 17, 2020.
[b] Since the end of in-person instruction on March 17, 2020.

© 2021 Raviv T et al. *JAMA Network Open.*

eTable 1 (continued).

ANNUAL HOUSEHOLD INCOME					
**$35,000-$49,999, N. (%)**		**$20,000-$34,999, N. (%)**		**<$20,000, N. (%)**	
**Before[a]**	**Since[b]**	**Before[a]**	**Since[b]**	**Before[a]**	**Since[b]**
201(4.9)	706(17.2)	263(5.4)	695(14.3)	288(7.5)	519(13.6)
465(11.3)	746(18.2)	463(9.5)	760(15.6)	403(10.5)	511(13.3)
153(3.7)	430(10.5)	177(3.6)	382(7.9)	176(4.6)	299(7.8)
162(3.9)	903(22)	191(3.9)	865(17.8)	191(5)	600(15.7)
484(11.8)	833(20.3)	560(11.5)	854(17.6)	489(12.8)	572(14.9)
32(0.8)	15(0.4)	18(0.4)	16(0.3)	19(0.5)	15(0.4)
2369(57.7)	1364(33.2)	2386(49)	1496(30.7)	1651(43.1)	1062(27.7)
1831(44.6)	1283(31.3)	1920(39.5)	1475(30.3)	1346(35.1)	1160(30.3)
2194(53.5)	1777(43.3)	2258(46.4)	19(39.5)	1523(39.8)	1408(36.8)
2128(51.9)	1699(41.4)	2624(53.9)	22(44.7)	1916(50)	1736(45.3)
1560(38)	1258(30.7)	1660(34.1)	13(26.8)	1094(28.6)	904(23.6)

[a] Before the end of in-person instruction on March 17, 2020.
[b] Since the end of in-person instruction on March 17, 2020.

© 2021 Raviv T et al. *JAMA Network Open.*

eTable 2. Frequency of Caregiver-Reported Youth Mental Health Concerns and Positive Adjustment Characteristics Before and Since the End of In-person Instruction

	**Mental Health Concerns**					
	**Angry**	**Anxious**	**Depressed**	**Lonely**	**Self-harm or suicidal ideation**	**Stressed**
	**N (%)**					
Not reported as present either before or since in-person instruction was ended	29913 (73.3%)	28270 (69.2%)	34211 (83.8%)	27049 (66.2%)	40333 (99.0%)	27696 (67.8%)
Reported as present before, but not since in-person instruction was ended	1173 (2.9%)	3085 (7.6%)	911 (2.2%)	784 (1.9%)	134 (0.3%)	3186 (7.8%)
Reported as present since, but not before, in-person instruction was ended	9209 (22.6%)	7445 (18.2%)	5236 (12.8%)	12351 (30.2%)	197 (0.5%)	8365 (20.5%)
Reported as present both before and since in-person instruction was ended	539 (1.3%)	2052 (5.0%)	476 (1.2%)	668 (1.6%)	59 (0.1%)	1587 (3.9%)

© 2021 Raviv T et al. *JAMA Network Open.*

eTable 2 (continued).

Positive Adjustment Characteristics					
**Hopeful/ Positive**	**Talks about plans for the future**	**Interacts positively with siblings/ family members**	**Has positive social/peer relationships**	**Relaxed**	
**N (%)**					
17640 (43.2%)	22738 (55.7%)	13099 (32.1%)	12172 (29.8%)	15111 (37.0%)	
11200 (27.4%)	18114 (44.3%)	8623 (21.1%)	14294 (35.0%)	10685 (26.2%)	
3160 (7.7%)	2766 (6.8%)	3087 (7.6%)	1685 (4.1%)	4327 (10.6%)	
8852 (21.7%)	9824 (24.1%)	16043 (39.3%)	12701 (31.1%)	10729 (26.3%)	

© 2021 Raviv T et al. *JAMA Network Open.*

eTable 3. Covariate-Adjusted Probability of Parental Endorsement of Mental Health Concerns and Positive Adjustment Characteristics

	Mental Health Concerns					
	**Angry**	**Anxious**	**Depressed**	**Lonely**	**Self-harm or suicidal ideation**	**Stressed**
	**Odds Ratio, (95% Confidence Interval)**					
Characteristic Reported Pre-COVID (vs. unreported)	1.71 (1.50-1.96)	2.22 (2.06-2.40)	3.27 (2.82-3.78	1.94 (1.68-2.23)	83.9 (53.7-131.1)	1.53 (1.41-1.66)
Total COVID Exposure and Family Impact	1.55 (1.48-1.62)	1.56 (1.49-1.63)	1.52 (1.45-1.60)	1.42 (1.37-1.48)	1.40 (1.10-1.78)	1.52 (1.46-1.59)
**Geographic Region**	*p<.001*	*p=.21*	*p<.001*	*p<.001*	*p=.22*	*p=.04*
Central (vs. North)	1.00 (0.91-1.10)	1.00 (0.91-1.09)	0.92 (0.83-1.03)	0.98 (0.90-1.07)	1.53 (1.01-2.34)	0.97 (0.88-1.06)
South (vs. North)	0.91 (0.81-1.01)	0.96 (0.86-1.08)	0.88 (0.77-1.00)	0.86 (0.78-0.95)	1.21 (0.65-2.26)	0.97 (0.87-1.08)
Southwest (vs. North)	0.75 (0.69-0.82)	0.87 (0.80-0.95)	0.75 (0.68-0.84)	0.76 (0.70-0.82)	0.89 (0.56-1.41)	0.89 (0.82-0.97)
**Annual Household Income**	*p<.001*	*p<.001*	*p=.009*	*p<.001*	*p<.001*	*p<.001*
$75K-$99K (vs $100K+)	0.89 (0.82-0.98)	0.89 (0.82-0.98)	0.86 (0.77-0.96)	0.85 (0.78-0.92)	0.92 (0.57-1.47)	0.94 (0.86-1.03)
$50K-74K (vs. $100K+)	0.69 (0.63-0.76)	0.78 (0.71-0.86)	0.72 (0.64-0.81)	0.66 (0.60-0.72)	0.91 (0.55-1.47)	0.77 (0.70-0.85)
$35K-$49K (vs. $100K+)	0.52 (0.46-0.59)	0.58 (0.51-0.65)	0.61 (0.53-0.70)	0.48 (0.43-0.54)	0.46 (0.22-0.97)	0.64 (0.57-0.72)
$20K-$34K (vs. $100K+)	0.42 (0.37-0.47)	0.48 (0.43-0.54)	0.43 (0.37-0.50)	0.37 (0.33-0.41)	0.45 (0.24-0.86)	0.51 (0.45-.56)
<$20K (vs. $100K+)	0.38 (0.33-0.43)	0.38 (0.33-0.43)	0.43 (0.37-0.50)	0.30 (0.27-0.34)	0.34 (0.14-0.83)	0.41 (0.36-0.47)
**Race/Ethnicity**	*p<.001*	*p<.001*	*p<.001*	*p<.001*	*p<.001*	*p<.001*
Black (vs. White Race)	0.39 (0.35-0.44)	0.46 (0.41-0.51)	0.40 (0.35-0.46)	0.45 (0.41-0.50)	0.40 (0.21-0.74)	0.49 (0.44-0.55)
Latinx (vs. White Race)	0.42 (0.39-0.46)	0.56 (0.51-0.61)	0.40 (0.36-0.45)	0.33 (0.31-0.36)	0.38 (0.23-0.63)	0.57 (0.52-0.62)
Multi/Other (vs. White Race)	0.65 (0.58-0.72)	0.75 (0.67-0.84)	0.64 (0.56-0.73)	0.65 (0.59-0.72)	0.91 (0.55-1.51)	0.78 (0.70-0.87)

© 2021 Raviv T et al. *JAMA Network Open.*

eTable 3 (continued).

	Positive Adjustment Characteristics				
	**Hopeful/ Positive**	**Talks about plans for the future**	**Interacts positively with siblings/ family members**	**Has positive social/peer relationships**	**Relaxed**
	**Odds Ratio, (95% Confidence Interval)**				
Characteristic Reported Pre-COVID (vs. unreported)	4.85 (4.57-5.15)	8.72 (8.19-9.29)	7.97 (7.50-8.46)	6.37 (5.93-6.84)	3.58 (3.39-3.78)
Total COVID Exposure and Family Impact	0.88 (0.84-0.92)	0.92 (0.88-0.96)	0.86 (0.82-0.90)	0.87 (0.83-0.91)	0.79 (0.76-0.82)
**Geographic Region**	*p=.02*	*p=.002*	*p=.24*	*p=.09*	*p=.60*
North (vs. Central)	1.02 (0.93-1.12)	1.09 (0.99-1.20)	1.02 (0.93-1.11)	1.04 (0.95-1.13)	1.04 (0.95-1.14)
South (vs. Central)	1.08 (0.97-1.19)	1.06 (0.96-1.18)	1.16 (1.05-1.28)	1.13 (1.03-1.25)	1.06 (0.97-1.17)
Southwest (vs. Central)	1.13 (1.04-1.22)	1.01 (0.93-1.10)	1.07 (0.99-1.16)	1.05 (0.97-1.13)	1.11 (1.03-1.20)
**Annual Household Income**	*p<.001*	*p<.001*	*p<.001*	*p<.001*	*p<.001*
$75K-$99K (vs $100K+)	1.01 (0.92-1.11)	1.06 (0.96-1.17)	0.93 (0.85-1.02)	0.87 (0.79-0.95)	1.07 (0.98-1.17)
$50K-74K (vs. $100K+)	1.08 (0.98-1.20)	1.12 (1.01-1.23)	0.96 (0.87-1.05)	0.97 (0.88-1.06)	1.21 (1.11-1.33)
$35K-$49K (vs. $100K+)	1.16 (1.04-1.29)	1.16 (1.03-1.29)	0.92 (0.83-1.03)	0.98 (0.89-1.09)	1.38 (1.25-1.53)
$20K-$34K (vs. $100K+)	1.18 (1.06-1.30)	1.01 (0.90-1.13)	0.88 (0.80-0.98)	1.05 (0.95-1.17)	1.61 (1.46-1.77)
<$20K (vs. $100K+)	1.27 (1.14-1.42)	0.92 (0.82-1.04)	0.89 (0.81-1.00)	1.00 (0.89-1.11)	1.76 (1.59-1.96)
**Race/Ethnicity**	*p<.001*	*p<.001*	*p<.001*	*p<.001*	*p<.001*
Black (vs. White Race)	1.84 (1.66-2.03)	1.47 (1.33-1.63)	1.22 (1.11-1.34)	1.26 (1.15-1.39)	1.77 (1.61-1.94)
Latinx (vs. White Race)	1.67 (1.53-1.81)	1.15 (1.05-1.26)	1.11 (1.03-1.21)	1.14 (1.06-1.24)	1.66 (1.53-1.79)
Multi/Other (vs. White Race)	1.47 (1.31-1.64)	1.21 (1.08-1.37)	1.22 (1.09-1.36)	1.24 (1.11-1.38)	1.55 (1.39-1.73)

Note: All reported P values are from asymptotic likelihood ratio tests evaluating the joint significance of the included categorical variables (i.e., geographic region, annual household income (modeled as ordinal) and race/ethnicity vs. a model omitting that specific categorical variable.

© 2021 Raviv T et al. *JAMA Network Open.*

eTable 4. COVID-19 Exposure/Family Impacts by Household Income Level

	ANNUAL HOUSEHOLD INCOME					
	**>$100,000**	**$75,000-$99,999**	**$50,000-$74,999**	**$35,000-$49,999**	**$20,000-$34,999**	**<$20,000**
	**N (%)**					
Stopped working temporarily	776(8)	645(17.1)	898(22.3)	844(26.1)	1245(32.5)	956(32.2)
Permanently lost job	399(4.1)	279(7.4)	375(9.3)	321(9.9)	487(12.7)	507(17.1)
Kept working outside of home	3500(36.2)	1787(47.3)	2076(51.6)	1820(56.2)	1927(50.3)	1123(37.8)
Healthcare provider	1375(14.2)	543(14.4)	521(12.9)	372(11.5)	370(9.7)	231(7.8)
Cut back hours	1830(18.9)	796(21)	928(23.1)	861(26.6)	1187(31)	791(26.6)
Moved out of home	24(0.2)	16(0.4)	15(0.4)	27(0.8)	34(0.9)	55(1.8)
Lost health insurance	74(0.8)	70(1.9)	126(3.1)	96(3)	115(3)	81(2.7)
Family income decreased	2301(23.8)	1107(29.3)	1380(34.3)	1264(39)	1858(48.5)	1433(48.2)
Difficulty getting other essentials	413(4.3)	333(8.8)	568(14.1)	671(20.7)	911(23.8)	884(29.7)
Difficulty getting medicine	90(0.9)	78(2.1)	128(3.2)	145(4.5)	242(6.3)	279(9.4)
Difficulty getting healthcare	180(1.9)	162(4.3)	245(6.1)	267(8.2)	337(8.8)	321(10.8)
Difficulty getting food	113(1.2)	106(2.8)	214(5.3)	298(9.2)	419(10.9)	519(17.5)
Difficulty getting facemasks, sanitizer, or other products	1534(15.9)	966(25.5)	1292(32.1)	1267(39.1)	1673(43.7)	1464(49.2)
Couldn’t pay rent	79(0.8)	97(2.6)	218(5.4)	273(8.4)	451(11.8)	542(18.2)
Couldn't pay bills	91(0.9)	131(3.5)	284(7.1)	371(11.5)	636(16.6)	731(24.6)
Children took on job outside of home	23(0.2)	27(0.7)	32(0.8)	41(1.3)	63(1.6)	46(1.5)
Children assumed childcare responsibilities	448(4.6)	222(5.9)	309(7.7)	295(9.1)	299(7.5)	182(6.1)
Someone in family was exposed	1306(13.5)	642(17)	699(17.4)	507(15.7)	586(15.3)	368(12.4)
Someone in family had symptoms or was diagnosed	705(7.3)	394(10.4)	524(13)	428(13.2)	542(14.2)	366(12.3)
Someone in family passed away from COVID-19	225(2.3)	174(4.6)	212(5.3)	207(6.4)	220(5.7)	199(6.7)
	**Mean, (SD)**					
**Summary Overall COVID-19 Impact (possible range 0-20**)	1.6(1.6)	2.27(1.9)	2.74(2.2)	3.21(2.4)	3.55(2.5)	3.7(2.7)

© 2021 Raviv T et al. *JAMA Network Open.*
